# Evaluation of *Bacillus* Strains for Plant Growth Promotion and Predictability of Efficacy by *In Vitro* Physiological Traits

**DOI:** 10.1155/2018/5686874

**Published:** 2018-10-03

**Authors:** Rufus J. Akinrinlola, Gary Y. Yuen, Rhae A. Drijber, Anthony O. Adesemoye

**Affiliations:** ^1^Department of Plant Pathology, University of Nebraska-Lincoln, Lincoln, NE 68583, USA; ^2^Department of Agronomy and Horticulture, University of Nebraska-Lincoln, Lincoln, NE 68583, USA; ^3^West Central Research and Extension Center, University of Nebraska-Lincoln, 402 W. State Farm Road, North Platte, NE 69101, USA

## Abstract

Bacilli are commonly used as plant growth-promoting agents but can be limited in effectiveness to certain crop and soil environments. The objectives of this study were to (1) identify *Bacillus* strains that can be consistent in promoting the growth of corn, wheat, and soybean and (2) determine whether physiological traits expressed *in vitro* can be predictive of growth promotion efficacy/consistency and be used for selecting effective strains. Twelve *Bacillus* strains isolated from wheat rhizospheres were evaluated in greenhouse pot tests with nonsterile soil for their effects on the growth of corn, soybean, and wheat. The strains also were assessed *in vitro* for multiple physiological traits. All 12 strains increased corn growth significantly compared to the controls. The four most efficacious strains on corn—*Bacillus megaterium* R181, *B. safensis* R173, *B. simplex* R180, and *Paenibacillus graminis* R200—also increased the growth of soybean and wheat. No set of traits was a predictor of growth promotion efficacy. The number of traits expressed by a strain also was not an indicator of efficacy as strain R200 that was positive for only one trait showed high growth promotion efficacy. Effective strains can be identified through pot tests on multiple crop plants, but *in vitro* physiological assays are unreliable for strain selection.

## 1. Introduction

There is growing interest in the use of root-colonizing, plant growth-promoting rhizobacteria (PGPR) as supplements or alternatives to the use of chemicals to increase crop productivity in agriculture. Studies have shown that PGPR have great potentials to increase growth and/or yields of different crops. Crop yield increases because PGPR can be as high as 57%, depending on the crop [1–3].

Many bacterial genera have been utilized as PGPR, including *Agrobacterium*, *Arthrobacter*, *Azotobacter*, *Azospirillum*, *Bacillus*, *Burkholderia*, *Caulobacter*, *Chromobacterium*, *Erwinia*, *Flavobacterium*, *Micrococcus*, *Pseudomonas,* and *Serratia* [4]. Among these, members of the rod-shaped, endospore-forming Gram-positive *Bacillus* group are the most commonly commercialized [5]. This group includes bacteria previously classified in the genus *Bacillus* but now separated into different genera such as *Bacillus*, *Paenibacillus* and *Lysinibacillus* [6]. These bacteria are favored for commercialization as PGPR in part for their ability to produce heat and desiccation-tolerant endospores. These structures are critical in maintaining high cell viability and prolonging shelf life in formulations kept in storage [6]. Some *Bacillus* PGPR strains also have been reported to perform well under different environmental conditions [7, 8].

Although the potentials for using PGPR to improve crop production and increase yields are well recognized, the use PGPR is not yet a widespread practice in a large part because of inconsistency in plant growth promotion by most PGPR strains under different field conditions [9]. One factor that may be responsible for the inconsistency of a PGPR strain is its sensitivity to plant and soil conditions that limit its ability to colonize the rhizosphere and express growth promotion mechanisms; these conditions include soil type, temperature, moisture content, organic matter, and pH [7, 10, 11]. Because these conditions can vary considerably across different geographic locations, the effectiveness of PGPR is frequently reported to vary depending on the location in which they are applied. For example, Suslow et al. [12] reported that a strain of PGPR increased sugar beet yield in California field tests but failed consistently when tested in Idaho, whereas another strain that caused the greatest yield benefits in Idaho had no significant effect in California trials. Also, a commercial seed treatment product containing *Bacillus firmus* I-1582, a PGPR and nematode biocontrol strain isolated in Israel, had no effect on soybean yields and was ineffective in controlling soybean cyst nematode when tested in several locations in Nebraska [13]. While commercial success of a PGPR requires that it perform well under different environments, selection of such organisms continues to be a challenge.

PGPR increase plant growth via direct or indirect mechanisms [14]. Direct plant growth promotion occurs when PGPR increase plant growth by supplying growth factors such as nutrients and hormones to plants [15]. Examples of direct growth promotion mechanisms include nitrogen fixation [16]; phosphate solubilization or iron mobilization by microbial siderophores [17]; and provision of hormones such as indole acetic acid, cytokinin, and gibberellin [18–21]. Indirect plant growth-promotion occurs when PGPR increase plant growth by suppressing the growth or activity of plant pathogens and deleterious rhizosphere inhabiting microorganisms [22–26]. This can occur through the production of antibiotics and lytic enzymes, competition for nutrients, or induced systemic resistance against pathogens [4, 15, 27, 28]. Among these mechanisms or traits, it is unclear which individual trait or set of traits could be predictive of growth promotion and thus be used as criteria for selecting the best growth promoters among potential strains.

The research reported here is a part of the efforts at the University of Nebraska-Lincoln to develop PGPR for use in multiple field crops under the diverse cropping systems of Nebraska, as well as biological control agents for soilborne pathogens [29]. It has been suggested that PGPR strains isolated from a region are better adapted to conditions prevalent in that region and, thus, would be more effective when applied to fields in the same region [30]. Thus, bacteria were isolated from Nebraska plants for this effort. Because of the advantages of using *Bacillus* as PGPR, and little research has been conducted previously on *Bacillus* PGPR in Nebraska, this study focused on bacterial isolates in *Bacillus* group. In this study, twelve *Bacillus* strains, isolated from the rhizosphere of wheat grown in Nebraska, were assessed for growth-promotion potentials on corn, soybean, and wheat in the greenhouse, with the objective of identifying strains that can effectively and consistently promote the growth of multiple crop plants. The same strains were evaluated in laboratory tests for expression of physiological traits associated with plant growth promotion. The objective was to determine the relationship between physiological traits expressed *in vitro* and growth promotion efficacy. Screening of organisms for growth promotion potential using conventional greenhouse pot tests might be time-consuming when evaluating large numbers of candidate organisms. Identification of traits that are predictive of growth promotion efficacy might be useful in developing and/or improving screening strategies for effective PGPR strains.

## 2. Materials and Methods

### 2.1. Strains and General Bacteriological Methods

The twelve *Bacillus* strains investigated in this study were isolated from the roots of wheat plants grown near North Platte, Nebraska. The isolates were selected from our collection based on preliminary screening in a previous study and were identified via 16s rDNA using 27F/1492R primer set through the method described by Parikh et al. [29]. Sequencing was done at the Institute for Integrative Genome Biology, University of California, Riverside. Sequences were edited using the Seqbuilder and EditSeq modules of DNASTAR® Lasergene Software v14 (DNASTAR, Madison, WI). Nucleotides were compared to the NCBI database through the Basic Local Alignment Search Tool (BLAST) algorithm and the sequences were submitted to the GenBank. The test strains and other microorganisms used in this study are listed in Table 1. All bacterial strains were stored at −75°C in nutrient broth amended with 10% glycerol and were routinely cultured on tenth-strength tryptic soy agar (10% TSA). Cell suspensions used in generating lawn cultures or inoculating seed were prepared by harvesting cells which has been incubated on 10% TSA for 36 to 48 h at 28°C, and the cells were suspended in 10 mM sodium phosphate buffer (PB) at pH 6.0. A spectrophotometer was used to measure the absorbance (600 nm) of each cell suspension which was then diluted with sterile phosphate buffer to an absorbance level corresponding to 10^8^ cfu/mL.

### 2.2. Evaluation of Growth Promotion through Greenhouse Pot Tests

Three greenhouse pot experiments were conducted, the first evaluating all twelve *Bacillus* strains for the ability to promote growth of corn. The five most efficacious strains in the corn experiment were subsequently evaluated in separate experiments using soybean and wheat. Each experiment was performed at least three times.

Seeds of corn (sweetcorn Sugar Buns F1 se+, Johnny's Selected Seeds), wheat (Overland W5-52, Huskers Genetics), and soybean (Vikings 2265, Johnny's Selected Seeds) were surface disinfected by soaking in 2% commercial bleach solution for 3 minutes and rinsed with sterile distilled water 10 times. Seeds were air dried in a laminar airflow-hood and kept at 4°C for later use. Surface-disinfected corn and wheat seeds were treated with bacterial strains by soaking in cell suspensions for 60 minutes, while soybean seeds were soaked in cell suspensions for 30 minutes. The soaking periods were determined in preliminary experiments to maximize populations of the applied bacteria on the seed without negatively affecting seed germination. Seeds were soaked in sterile PB as the control. To determine the populations of cells adhering to the treated seeds after soaking, samples of the seeds were washed in sterile PB and the wash was used in dilution plating on 10% TSA. Populations of applied test strains on treated seed were typically around 10^7^ cfu/seed.

Treated seeds were sown into a nonpasteurized potting mix comprised of a loam soil and sand mixed in 2 to 1 ratio by volume. The same soil was used in all greenhouse experiments. Analysis of the potting mix showed that it was made up of 61% sand, 26% silt, and 13% clay and contained 1.0% organic matter, 4.1 parts-per-million (ppm) nitrate-nitrogen, 7.0 ppm bicarb phosphorus, and 161 ppm exchangeable potassium. One corn seed was sown per pot, three soybean seeds were sown per pot, and five wheat seeds were sown per pot. Plastic pots with a dimension of 13 cm diameter and 11 cm depth was used in all studies. There were five to eight replicate pots for each seed treatment in every experiment. Pots were arranged in a completely randomized design on a bench in a greenhouse where temperatures varied from 24°C (night) to 31°C (day). Each experiment lasted for 20 days during which pots were watered once a day without fertilization. At the end of the experiment, soil was carefully washed off the plant roots under low running tap water, and the shoots and roots were separated. Shoot height, fresh and dry shoots, and root weights were measured. Dry weights were determined after drying for 3 days at 70°C.

### 2.3. Statistical Analysis of Greenhouse Experiments

Two procedures were applied to analyze growth parameter data (i.e., shoot height and shoot and root weights) using SAS software (SAS Institute, Cary, NC). Data from each experiment trial were analyzed separately. Dunnett's test was used to determine whether a growth variable measurement of a bacterial treatment was significantly different (*P* ≤ 0.05) from the control. To compare bacterial treatments with each other, analysis of variance (ANOVA) was conducted first to determine if there was a significant treatment effect within an experiment trial, and then mean separation was performed using Fisher's LSD test (*α* = 0.05) when a significant treatment effect was found in the ANOVA. In preliminary analysis of dry and wet biomass data, within-treatment variability was found to be lower for fresh biomass measurements compared to dry biomass measurements. Therefore, only fresh biomass measurements were analyzed further and are reported here.

Growth parameter data were used in calculating three values that were not subjected to statistical analysis. The first, “percent growth increase” (PGI), denotes the amount to which a strain increased a growth variable relative to the control in an experiment trial. It was calculated using the following equation:

(1) $$\begin{array}{c} \text{PGI }=\;\frac{Mt\;-\;Mc}{Mc}\;\times \;100, \end{array}$$

where *M*t and *M*c are the mean measurements of the treatment and the control, respectively. Another value, “growth stimulation frequency” (GSF), denotes how consistently a strain increased growth across trials of an experiment. GSF is expressed as the percentage of all cases (i.e., growth parameters across all trials of an experiment) in which a strain yielded a significant increase (95% confidence level) compared to the control. The third value, “frequency in top 3” (FIT3), was determined in the corn experiment, while a comparable value, “frequency in top 2” (FIT2), was determined in the soybean and wheat experiments. FIT3 and FIT2 are the percentages of all cases in repeated experiments in which a strain was among the three top strains and two top strains, respectively, in respect to the magnitude of growth variable measurements. In the corn experiments, the top three strains reflected the top quartile of the 12 strains tested. In the soybean and wheat experiments, only the top two strains were identified, which represented roughly the top third because fewer bacterial strains were evaluated.

### 2.4. *In Vitro* Tests for Indirect Growth Promotion Traits

The 12 *Bacillus* strains were evaluated for these traits associated with indirect growth promotion: antagonism against bacteria and fungi (i.e., true fungi and oomycetes); proteolytic and chitinolytic enzyme activities; and biosurfactant and siderophore production.

Antagonism against three phytopathogenic bacteria (*Clavibacter michiganensis* subsp. *nebraskensis*, *Xanthomonas campestris* pv. *phaseoli*, and *Pectobacterium carotovorum*) was evaluated on 10% TSA and nutrient agar (NA). The pathogens were selected (1) to represent diverse bacterial pathogens and (2) because of their relevance in Nebraska to the crops being evaluated. For example, *Clavibacter michiganensis* subsp. *nebraskensis* causes Goss's bacterial wilt and leaf blight, which is a major disease of corn in Nebraska and Midwest United States. Lawn cultures were generated for each bacterial pathogen by spreading 0.5 mL cell suspensions onto the surfaces of agar plates and then allowing the agar surfaces to air-dry aseptically in a transfer hood. Five 3 mm diameter wells were made in the agar of each plate using a sterile cork-borer. Each well was filled with 15 *µ*L cell suspension of a test *Bacillus* strain or sterile PB (negative control). *Bacillus amyloliquefaciens* IN937a was used as the positive control. Each *Bacillus* test strain was tested against each of the three bacterial pathogens on three plates of each medium. The plates were kept in the transfer hood for 15 min to allow absorption of the suspensions into the medium before incubation at 28°C for 2 days. Presence of a clear halo zone around a well was an indication of antagonism by the test strain against the bacterial pathogen. A test strain was considered to have antibacterial activity if it inhibited the growth of any one of the bacterial pathogens on any medium.

Antagonism against two phytopathogenic fungi (*Fusarium graminearum* and *Rhizoctonia solani)* and two oomycetes (*Pythium ultimum* and *P. irregulare*) was evaluated on 10% TSA and potato dextrose agar (PDA). These pathogens are important fungal pathogens to the three crops used for this study. The center of each agar plate was inoculated with a 3 mm diameter fungal plug cut with a sterilized cork-borer from a 3-day-old PDA culture of a test fungus or oomycete. Each plate was coinoculated with *B. amyloliquefaciens* KPS46 (positive control), sterile PB (negative control), and three test *Bacillus* strains using sterile toothpicks and incubated for 3 days at 25°C. Presence of a zone of hyphal growth inhibition around a bacterial colony was an indication of antagonism of the strain against the mycelial organism. A test strain was considered to have antifungal activity if it inhibited the growth of any fungus or oomycete on any medium.

*Bacillus* test strains were evaluated for protease and chitinase activities using indicator media containing the respective substrates. Protease activity was evaluated using a milk agar medium containing (g/L) powdered milk (10), yeast extract (0.5), ammonium sulfate (0.5), calcium chloride (0.5), potassium phosphate monobasic (0.1), potassium phosphate dibasic (0.1), and agar (18) and pH adjusted to 7.0 ± 0.2. Strain AP-209 of *B. mojavensis* was used as the positive control. Chitinase activity was evaluated on colloidal chitin medium as described by Abirami et al. [31] with *Lysobacter enzymogenes* C3 used as the positive control. In both assays, loopfuls of test strains and the positive control were applied to three plates of the indicator medium. The plates were incubated at 28°C for 2 or 5 days for the protease and chitinase assay, respectively, and then examined for zones of clearing around bacterial colonies as indication of enzymatic digestion of the substrate.

Biosurfactant activity was assessed using the method described by Kobayashi and Yuen [32]. Three 50 *µ*L droplets of fluid from a 2-day old tryptic soy broth (TSB) culture of each test strain were applied to the surface of Parafilm. Culture fluid of *Lysobacter enzymogenes* C3 and sterile TSB were used as positive and negative controls, respectively. Droplet diameters were measured; a droplet diameter greater than the negative control was an indication of droplet spread due to the presence of a biosurfactant.

Siderophore production was detected using the Chrome Azurol S (CAS) siderophore assay [33]. Each test strain was spot inoculated onto three plates of the medium using a sterile inoculating loop and incubated for 5 days at 28°C. Culture plates were flooded with 1 mL CAS solution. Plates were inoculated with *Serratia marcescens* 94A-429 as the positive control. A blue to pink color change in the agar under and around a bacterial colony within 30 min of flooding with CAS solution was an indication of siderophore production by the bacterium.

### 2.5. *In Vitro* Tests for Direct Growth Promotion Traits

The *Bacillus* strains were evaluated for these traits associated with, or indicative of direct growth promotion: phosphate solubilization, nitrogen fixation, indole acetic acid (IAA) production, and promotion of corn growth in a semisterile environment (growth pouches).

Phosphate solubilization activity was evaluated on Pikovskaya's agar medium [34]. Three plates of the medium were inoculated with a loopful of a test strain, and plates were inoculated with *Serratia marcescens* 94A-429 as the positive control. The plates were incubated at 28°C for 7 days and then examined for zones of clearing around the bacterial colonies indicative of phosphate solubilization activity.

Nitrogen fixation activity was evaluated on glucose nitrogen-free mineral (GNFM) agar medium using bromothymol blue (BTB) as an indicator [35]. The BTB was prepared by dissolving 0.5 g BTB into 100 mL distilled water and filter-sterilized. Each test strain was inoculated onto three GNFM plates. *Azospirillum brasilense* 99B-817 was used as positive control. Plates were incubated at 28 °C for 7 days, flooded with BTB solution, and then examined for color change in the agar from green to dark blue or bluish green as an indication of nitrogen fixation activity.

Indole acetic acid production was evaluated using Salkowski's reagent and nutrient broth supplemented with 0.5 g/L L-tryptophan [36]. Each test strain was first cultured in 10 mL 10% TSB for 1 day at 28°C, and then 2 mL of the culture was transferred into 20 mL tryptophan-supplemented nutrient broth. *Lysinibacillus macroides* AP-282 was used as the positive control, and *Lysobacter enzymogenes* C3 and sterile NB were used as negative controls. The cultures were incubated at 28°C for 6 days and centrifuged at 13000 × g for 15 min. One mL of culture supernatant was mixed with 2 mL Salkowski's reagent with a drop of orthophosphoric acid. The mixture was incubated in the dark for 30 min and then examined for the development of pink color as an indication of indole acetic acid production.

Direct growth promotion activity in a semisterile, soil-less environment was evaluated using surface-disinfected seeds of corn (sweetcorn Sugar Buns F1 se+) sown in growth pouches (Mega International, Minnesota). The growth pouch is a plastic bag lined with paper towel material that was sterilized before use. The seeds were surface disinfected and treated with strains as performed for the greenhouse test. Seeds treated with sterile PB were used as the negative control. Treated seeds were sown 3 seeds per pouch, and seven replicate pouches were made for each treatment. The pouches were watered with 10 mL sterile distilled water every other day and kept at room temperature under 16 h light and 8 h dark for 10 days. At the end of the experiment, shoots and roots were separated. Shoot fresh weight, shoot height, and root length were measured, and numbers of lateral roots were counted. The experiment was conducted three times. In each trial of the experiment, Dunnett's test was used to determine whether a bacterial treatment was significantly different (*P* ≤ 0.05) from the control. A strain was considered positive for growth promotion if it increased the same growth parameter in two or more trials or increased two or more growth parameters in the same trial.

## 3. Results

### 3.1. Growth Promotion Effects in Greenhouse Pot Tests

#### 3.1.1. Response of Corn to Test Strains

All the 12 *Bacillus* strains enhanced corn growth compared to the control (Table 2). Using Dunnett's test to compare individual strains with the control, each of the strains caused a significant increase of one or more growth variables in at least two trials (Table 2). *Bacillus simplex* R180 showed the highest growth stimulation frequency (GSF) of 100%, that is, it increased all growth parameters across trials, followed by *B. safensis* R176 (GSF of 83%) and *B. megaterium* R181 (GSF of 78%). Other strains induced growth stimulation in less than 70% of the growth parameters across all trials. Bacterial treatments had dramatic considerable effects on shoot and root weights, with the highest increases exceeding 200%. Effects on shoot height were much lower, with percent increases being less than 60%. There was large variation between trials in the percentage growth increase for all strains. For example, shoot height increases by strain R181 ranged from 18 to 45%, shoot weight increases ranged from 40 to 140%, and root weight increases ranged from 32 to 136%. There also was considerable within-treatment variability for fresh root measurements in trial 3 such that for some bacterial treatments, in which increases in root weight compared to the control exceeded 100%, the difference from the control was not statistically significant at the 95% confidence level.

Significant treatment effects were found through ANOVA tests in 6 out of 9 growth parameters (Table 3). Significant differences among strains, as indicated by the LSD test, occurred in 5 of the 6 growth parameters in which a significant treatment effect occurred (Table 3). Strains R181, R180, and R200 were found most often among the top 3 strains having FIT3 of 56, 50, and 50%, respectively. Strains R176, R190, and R198 did not appear among top 3 strains in any of the measurements. There was no significant difference among the top 3 strains for most growth parameters. The only one exception was a higher root biomass for strain R181 compared to the other strains in trial 1 (Table 3).

The four most effective strains—R177, R180, R181, and R200—from the corn growth promotion experiments, based on highest GSF and FIT3 (Tables 2 and 3), were selected for further evaluation on wheat and soybean. Although *B. safensis* R176 had a relatively high GSF of 83% (Table 2), it was not selected because it did not appear among the top 3 strains for any growth parameter (Table 3). Instead, *B. safensis* R173 was selected to represent the species in the experiments on soybean and wheat.

#### 3.1.2. Responses of Soybean and Wheat to Five Strains

In the soybean experiment trials, four strains—R173, R180, R181, and R200—induced significant growth increases compared to the control (Table 4). Each of these strains caused significant increases in root and shoot compared to the control, as indicated by Dunnett's test or the LSD test, in one or more trials of the experiment (Table 4). *Bacillus safensis* R173 had the highest GSF of 88%, whereas the GSF for the other three strains did not exceed 50%. The strains had greater effects on root growth than shoot growth, with the percent increases for root growth exceeding 90% in many cases, whereas percent increases for shoot growth were less than 50% (Table 4). The highest increases in shoot weight (46%) and root weight (144%) were lower than that found in the experiments with corn. None of the strains induced a significant increase in shoot height (data not shown). Among the four strains, R173, R180, and R181 were most frequently found in the top 2 strains category with FIT2 values of 100, 50, and 50%, respectively, whereas strain R200 had a relatively low FIT2 value of 25% (Table 4). There was no significant difference between the top 2 strains for any growth parameter (Table 4).

The same four strains that were positive for growth promotion on soybean (R173, R180, R181, and R200) also increased the growth of wheat compared to the control (Table 5), each strain causing a significant increase of two or more growth variables in two or more trials compared to the control according to Dunnett's or LSD tests. Strains R181 and R200 had a GSF value of 62%, while GSF values for R180 and R173 were 33 and 25%, respectively. Growth promotion was higher for root growth than for shoot growth, with increases in root weight induced by the four strains ranging from 43 to 115%, whereas shoot weight increases did not exceed 50%. These values were lower than that found in the corn experiments but similar to results found in the soybean experiments. Strains R181 and R200 were most consistently found among the top 2 strains with FIT2 of 75 and 63%, respectively (Table 5). There was no significant difference among the top 2 strains for any growth parameter (Table 5).

These results showed that four *Bacillus* strains (*B. safensis* R173, *B. simplex* R180, *B. megaterium* R181, and *P. graminis* R200) were effective for promoting soybean and wheat growth in greenhouse pot experiments. This indicated that these *Bacillus* strains have broad spectrum plant growth-promotion activity.

### 3.2. *In Vitro* Tests for Indirect Growth Promotion Traits

The results of the *in vitro* assays are summarized in Table 6 Only three strains—*B. megaterium* R181 and *B. pumilus* strains R183 and R190—exhibited antagonism against phytopathogenic bacteria (Table 6). Each of these strains was inhibitory to either *Clavibacter michiganensis* subsp. *nebraskensis* or *Xanthomonas campestris*, but not both, and none inhibited *Pectobacterium carotovorum* (data not shown). The same three strains, in addition to *B. pumilus* R174, exhibited antagonism against fungi (Table 6). That activity was limited to a transitory inhibition against *F. graminearum,* while *R. solani* and the two *Pythium* spp. were unaffected (data not shown).

Nine out of the twelve test strains were positive for protease activity on milk agar medium (Table 6) including the three strains (*B. pumilus* R183 and R190, and *B. megaterium* R181) that exhibited antibacterial activity. In contrast, none of the test strains exhibited chitinase activity (Table 6). All strains of *B. pumilus* and *B. safensis* were positive for biosurfactant activity (Table 6. Four of the *Bacillus* strains (R180, R181, R190, and R232) were positive for siderophore production on CAS agar medium (Table 6).

### 3.3. *In Vitro* Tests for Direct Growth Promotion Traits

Strains R173 and R177 of *B. safensis* and strains R181 and R232 of *B. megaterium* exhibited phosphate solubilization on Pikovskaya's agar medium (Table 6). None of the twelve test strains was found to exhibit nitrogen fixation activity (Table 6). Seven of the twelve test strains, including all strains of *B. megaterium* and *B. safensis*, were found to produce indole acetic acid (Table 6). None of the three strains of *B. pumilus*, however, exhibited this activity.

Nine of the twelve strains exhibited the potential to increase plant growth under semisterile conditions in growth pouches, increasing a single growth parameter compared to the control in at least two trials or multiple growth parameters in a single trial (Table 7).

### 3.4. Relationship of Physiological Traits to Growth Promotion Efficacy

*Bacillus* test strains are listed in Table 6 according to growth promotion efficacy demonstrated in the greenhouse corn experiments, along with each strain's profile of *in vitro* physiological traits. There was no individual specific trait or group of traits that clearly distinguished the high growth promotion efficacy group from the low-efficacy group. There also appeared to be no relationship between numbers of physiological traits and effectiveness in growth promotion. The highest number of traits was exhibited by *B. megaterium* R181 in the high-efficacy group, but the lowest number of traits also was found in a member of the high-efficacy group, *P. graminis* R200.

## 4. Discussion

All of the 12 *Bacillus* strains tested in this study exhibited the potential to increase plant growth in the corn experiments. Four strains—*B. megaterium* R181, *B. safensis* R173, *B. simplex* R180, and *P. graminis* R200—were shown to have broad-spectrum activity as they were effective in increasing the growth of soybean and wheat as well. Growth promotion of all three crop plants by *Bacillus megaterium* R181 in this study agrees with previous studies that demonstrated *B. megaterium* strains being effective for growth promotion on a variety of crop plants [19, 37]. Our findings with strains R173, R180, and R181 set new precedencies for the species represented by the strains. First, *B. safensis* was reported previously to increase plant growth on corn [38]; our study expands the range of crop plants that can be stimulated by *B. safensis* to include soybean and wheat. Second, while growth promotion by strains of *B. simplex* was demonstrated previously on kiwifruit [39], pea [40], strawberry [41], and tomato [42], our study is the first to demonstrate that a strain of *B. simplex* can increase growth in corn and soybean. Third, strains of *P. graminis* were reported to exhibit growth promotion-associated traits *in vitro* such as nitrogen fixation and extracellular enzyme production [43], but this is the first demonstration of a *P. graminis* strain having plant growth promotion ability.

We found that corn was more responsive in general to the *Bacillus* strains than soybean or wheat, and this finding is in line with other studies. For example, Tilak and Reddy [44] reported that strains of *Bacillus circulans* and *B. cereus* increased growth in corn, wheat, and pigeon pea, but the highest response to the bacterial treatments was found in corn. In another study, Khalid et al. [45] evaluated thirty bacterial strains' plant growth promotion on wheat seedlings and found only four to be effective.

We observed trial-to-trial variation in growth promotion by every *Bacillus* strain. Such variability has been reported in other growth promotion studies and could be due to variations in many edaphic and host factors [7, 10, 11]. For example, Cakmakçi et al. [10] reported that variability in plant growth responses to bacterial inoculation was partly due to changes in soil organic matter content. But the pot experiments in our study were conducted using the same potting medium and efforts were made to maintain uniform moisture condition, while greenhouse conditions were controlled to minimize seasonal temperature changes. Thus, we cannot point to any obvious environmental condition that could explain the variability we observed. The broad-spectrum strains identified in this study were more consistent in activity between trials than the other strains, suggesting that they might be more tolerant to variations in soil conditions. The effectiveness of these broad-spectrum strains in different field environments, however, needs to be determined. The results from testing of *in vitro* physiological traits associated with direct and indirect growth promotion provide clues as to the mechanisms by which individual *Bacillus* strains can enhance the growth of corn. Direct growth promotion appeared to be the most common mode of action as 10 of the 12 strains exhibited the ability to promote corn growth in growth pouches or some combination of growth promotion in growth pouches, phosphate solubilization activity, and IAA production. In contrast, growth promotion by *B. pumilus* appears to involve indirect mechanisms primarily as all three strains exhibited antibacterial and antifungal antagonism, as well as combinations of siderophore, biosurfactant, and protease activities *in vitro*.

When *in vitro* physiological traits are examined relative to effectiveness in growth promotion, there was no individual specific trait or group of traits that clearly distinguished the high growth promotion efficacy group from the low-efficacy group. Four traits—protease, biosurfactant, antibacterial, and antifungal inhibition—were found more commonly among the low-efficacy strains than the high-efficacy strains (Table 7). Indole acetic acid production and phosphate solubilization are two traits that were more common among strains in the high-efficacy group than strains in the low-efficacy group, but these traits were absent from two high-efficacy strains, R180 and R200. Based on these findings, we consider indole acetic acid production and phosphate solubilization to be traits that might contribute to high growth promotion efficacy, but their expression is not predictive of growth promotion efficacy. The relationship between physiological traits and effectiveness in growth promotion also was examined from the perspective of the number of physiological traits exhibited by strains in the high- and low-efficacy categories. Expression of numerous traits by a strain was not always consistent with exhibition of high plant growth-promotion efficacy. For example, *B. pumilus* R190, which was in the low-efficacy group in terms of plant growth promotion, exhibited five out of the 10 traits, a higher number than all of the high-efficacy strains except *B. megaterium* R181 (Table 7). Conversely, *P. graminis* R200, which was in the high-efficacy group, exhibited only the ability to promote growth in a growth pouch. In summary, while the specific physiological traits might contribute to growth promotion activity, the relationship between physiological traits in general and growth promotion efficacy remains unclear.

There are three main conclusions from this study. First, a high percentage of strains tested in this study, representing diverse species, were found to be effective on corn, and some were effective on multiple crop plants. This suggests that that highly diverse populations of beneficial plant growth-promoting bacilli are indigenous to the U.S. Great Plains region and could be explored as biological tool for sustainable crop production. Second, while variability in effectiveness occurs in all PGPR strains, growth stimulation frequency and frequency in the top two or three strains in repeated trials are useful parameters in selecting effective strains that have reduced variability in effectiveness. Third, the effectiveness of a PGPR strain in promoting plant growth in a soil environment cannot be reliably predicted by any one or a group of physiological traits. Although indole acetic acid production and phosphate solubilization might contribute to growth promotion in certain strains, other mechanisms might be important in other strains. Given that no individual physiological traits are predictive of effectiveness in growth promotion, we do not recommend testing of physiological traits as the first criterion for selecting effective plant growth promoters. Greenhouse pot tests are a more effective and more direct screening method to identify effective strains. Effective growth promoter strains from pot tests can then be tested for their physiological traits to determine their modes of action. Knowledge of mode of action then could be used to better match strains with their intended use, for example, using direct growth promotion strains in nutrient deficient soils and indirect growth promotion strains in soils with high populations of deleterious microbes.

## Figures and Tables

**Table 1:** Microorganisms used in this study.

Organism	Purpose/accession number	Source
*Bacillus acidiceler* R228	Test strain/KY515411	Identified in this study
*B. megaterium* R181	Test strain/KY807994	
*B. megaterium* R232	Test strain/KY515414	
*B. pumilus* R174	Test strain/KY515394	
*B. pumilus* R183	Test strain/KY515399	
*B. pumilus* R190	Test strain/KY515404	
*B. safensis* R173	Test strain/KY515393	
*B. safensis* R176	Test strain/KY515395	
*B. simplex* R180	Test strain/KY515398	
*Lysinibacillus fusiformis* R198	Test strain/KY515408	
*Paenibacillus cineris* R177	Test strain/KY515396	
*P. graminis* R200	Test strain/KY515409	

*Azospirillum brasilense* 99B-817	Positive control for nitrogen fixation assay	Dr. Joseph Kloepper, Auburn University, Alabama
*B. mojavensis* AP-209	Positive control for protease enzyme assay	
*B. amyloliquefaciens* IN937A	Positive control for bacterial inhibition assay	
*Lysinibacillus macroides* AP-282	Positive control for IAA assay.	
*Serratia marcescens* 94A-429	Positive control for siderophore and phosphate solubilization assays	

*B. amyloliquefaciens* KPS46	Positive control for fungal inhibition assay	Dr. Gary Yuen, University of Nebraska-Lincoln
*Lysobacter enzymogenes* C3	Positive control for biosurfactant and chitinase assays	
*Clavibacter michiganensis* subsp. *nebraskensis*	Plant pathogenic bacteria for inhibition assay	
*Pectobacterium carotovorum*	Plant pathogenic bacterium for inhibition assay	
*Xanthomonas campestris* pv. *phaseoli*	Plant pathogenic bacterium for inhibition assay	
*Fusarium graminearum* PH-1	Plant pathogenic fungus for inhibition assay	
*Rhizoctonia solani* R251	Plant pathogenic fungus for inhibition assay	
*Pythium irregulare*	Plant pathogenic oomycete for inhibition assay	
*Pythium ultimum*	Plant pathogenic oomycete for inhibition assay	

**Table 2:** Promotion of corn shoot and root growth by twelve *Bacillus* strains in three greenhouse pot experiments.

Strain	% increase compared to control^w^									GSF (%)^x^
	Shoot height			Shoot fresh weight			Root fresh weight			
	Trial 1	Trial 2	Trial 3	Trial 1	Trial 2	Trial 3	Trial 1	Trial 2	Trial 3	
*Bacillus acidiceler* R228	7	42^*∗*^	28^*∗*^	7	118^*∗*^	66	−11	155^*∗*^	92	44
*B. megaterium* R181	19^*∗*^	45^*∗*^	28^*∗*^	40	140^*∗*^	59^*∗*^	36^*∗*^	121^*∗*^	132	78
*B. megaterium* R232	17	45^*∗*^	12	24	144^*∗*^	32^*∗*^	—6	107^*∗*^	173	44
*B. pumilus* R174	13	41^*∗*^	7	30	126^*∗*^	24	—3	117^*∗*^	91	33
*B. pumilus* R183	12	40^*∗*^	28^*∗*^	33	103^*∗*^	62	0	122^*∗*^	12	44
*B. pumilus* R190	—	38^*∗*^	13	—	77^*∗*^	32	—	93^*∗*^	104	50
*B. safensis* R173	3	44^*∗*^	15^*∗*^	−15	137^*∗*^	51^*∗*^	−14	167^*∗*^	222	56
*B. safensis* R176	—	34^*∗*^	20^*∗*^	—	111^*∗*^	42^*∗*^	—	124^*∗*^	110	83
*B. simplex* R180	—	41^*∗*^	30^*∗*^	—	118^*∗*^	68^*∗*^	—	112^*∗*^	206^*∗*^	100
*Lysinibacillus fusiformis* R198	5	47^*∗*^	17^*∗*^	6	122^*∗*^	33^*∗*^	−25	135^*∗*^	147^*∗*^	56
*Paenibacillus cineris* R177	9	51^*∗*^	20^*∗*^	20	155^*∗*^	42	3	168^*∗*^	−8	67
*P. graminis* R200	—	54^*∗*^	18^*∗*^	—	215^*∗*^	37	—	203^*∗*^	75	67

^w^Percentage increase of a growth variable by bacterial treatment compared to the control. ^x^GSF = growth stimulation frequency; frequency at which a strain increased growth (at 95% confidence level) in all measurements across trials. ^*∗*^Significant difference between treatment and control (*P* ≤ 0.05) according to Dunnett's test. — = no data because strain was not tested.

**Table 3:** Comparison of twelve *Bacillus* strains for effects on corn shoot and root growth in greenhouse pot experiments, with the three strains having the highest measurements for each parameter indicated with shading.

Strain	Shoot height (cm)			Shoot fresh weight (g)			Root fresh weight (g)			FIT3 (%)^w^
	Trial 1	Trial 2	Trial 3	Trial 1	Trial 2	Trial 3	Trial 1	Trial 2	Trial 3	
*Bacillus acidiceler* R228	46	47ba^x^	40a	6.0	6.0bc	3.2ab	3.2b	3.5ab	0.9	22
*B. megaterium* R181	51	48ab	40a	8.0	6.5abc	3.0ab	4.9a	3.2ab	1.1	56
*B. megaterium* R232	50	48ab	35cde	7.0	6.6abc	2.5abcd	3.4b	3.1b	1.3	44
*B. pumilus* R174	49	47b	33cde	7.0	6.0bc	2.4bcd	3.5b	3.1b	0.9	11
*B. pumilus* R183	48	47b	40ab	7.0	5.5bc	3.1ab	3.6b	3.2ab	1.1	33
*B. pumilus* R190	—	46b	35bcde	—	4.8c	2.5abcd	—	2.8b	1.0	0
*B. safensis* R173	44	48ab	36abcd	6.0	6.4bc	2.9ab	3.1b	3.9ab	1.6	22
*B. safensis* R176	—	47b	37abc	—	5.7bc	2.7abc	—	3.3ab	1.0	0
*B. simplex* R180	—	47b	41a	—	5.9bc	3.2a	—	3.2b	1.5	50
*Lysinibacillus fusiformis* R198	45	48ab	36abcd	6.0	6.0bc	2.5abcd	2.7b	3.5ab	1.2	0
*Paenibacillus cineris* R177	47	50ab	37abc	7.0	6.9ab	2.7abcd	3.7b	3.9ab	0.5	44
*P. graminis* R200	—	53a	37abcd	—	8.4a	2.6abcd	—	4.3a	0.9	50
Control	43	33c	31e	5	2.7d	1.9d	3.6b	1.5c	0.5	NA^y^
ANOVA P	0.081	<0.001	0.002	0.224	0.001	0.042	0.014	0.005	0.614	NA

^w^FIT3 = frequency in top 3 strains category. ^x^Numbers followed by the same letter in each column are not significantly different at *α* = 0.05 according to LSD test. ^y^NA = Not applicable. — = no data or strain was not tested.

**Table 4:** Growth promotion effects of five *Bacillus* strains on soybean plants in 4 greenhouse pot experiments, with the two strains having the highest measurements for each parameter indicated with shading.

Strain	Shoot fresh weight (g) (% increase)^t^				Root fresh weight (g) (% increase)^t^				GSF (%)^u^	FIT2 (%)^v^
	Trial 1	Trial 2	Trial 3	Trial 4	Trial 1	Trial 2	Trial 3	Trial 4		
*Bacillus megaterium* R181	1.3bc^w^ (18)	1.3ab (18)	3.7ab^*∗*^ (16)	3.8ab (3)	0.42b (−9)	0.64b (60)	1.7b^*∗*^ (89)	2.1a (31)	50	50
*B. safensis* R173	1.5ab^*∗*^ (36)	1.5a^*∗*^ (36)	4.2a^*∗*^ (31)	4.2a (14)	0.62ab (35)	0.97a^*∗*^ (142)	2.2a^*∗*^ (144)	2.1a (31)	88	100
*B. simplex* R180	—	—	3.9a^*∗*^ (22)	3.1b (−16)	—	—	2.2a^*∗*^ (144)	1.3c (−19)	50	50
*Paenibacillus cineris* R177	1.1c (0)	1.1c (0)	—	—	0.38b (−17)	0.35c (−13)	—	—	0	0
*P. graminis* R200	1.6a^*∗*^ (46)	1.2bc (9)	3.5ab (9)	3.6ab (−3)	0.88a^*∗*^ (91)	0.41c (3)	1.5b^*∗*^ (67)	1.8ab (13)	38	25
Control	1.1c	1.1bc	3.2ab	3.7b	0.46b	0.40c	0.9c	1.6bc	NA^x^	NA
ANOVA P	0.001	0.004	0.054	0.059	0.003	<0.001	<0.001	0.004	NA	NA

^t^Percentage increase of a growth variable by bacterial treatment compared to the control. ^u^GSF = growth stimulation frequency; frequency at which a strain increased growth (at 95% confidence level) in all measurements across trials. ^v^FIT2  = Frequency in top 2 strains category. ^w^Numbers followed by the same letter in each column are not significantly different at *α* = 0.05 according to LSD test. ^x^NA = not applicable. ^*∗*^Significant difference between treatment and control (*P* ≤ 0.05) according to Dunnett's test. — = no data or strain was not tested.

**Table 5:** Growth promotion effects of five *Bacillus* strains on wheat plants in 3^w^ greenhouse pot experiments, with the two strains having the highest measurements for each parameter indicated with shading.

Strain	Shoot height (cm) (% increase)^t^		Shoot fresh weight (g) (% increase)					Root fresh weight (% increase)	GSF (%)^u^	FIT2 (%)^v^
	Trial 1	Trial 2	Trial 1	Trial 2	Trial 3	Trial 1	Trial 2	Trial 3		
*Bacillus megaterium* R181	38a^*∗*^^w^ (15)	35^*∗*^ (21)	0.44a^*∗*^ (47)	0.13 (30)	0.46 (7)	0.33a^*∗*^ (154)	0.36a (29)	0.10a (43)	62	75
*B. safensis* R173	36ab (9)	31 (7)	0.42a^*∗*^ (40)	0.11 (10)	0.54 (26)	0.29ab^*∗*^ (123)	0.27ab (−4)	0.08ab (14)	25	38
*B. simplex* R180	37a (12)	33 (14)	0.31b (3)	0.13 (30)	0.59^*∗*^ (37)	—	—	0.07b (0)	33	33
*Paenibacillus cineris* R177	—	—	—	—	—	0.20bc (54)	0.24b (−14)	—	0	0
*P. graminis* R200	38a^*∗*^ (15)	33 (14)	0.43a^*∗*^ (43)	0.13^*∗*^ (30)	0.49 (14)	0.28ab^*∗*^ (115)	0.18b (−36)	0.11a^*∗*^ (57)	62	62
Control	33b	29	0.30b	0.10	0.43	0.13c	0.28ab	0.07b	NA^x^	NA
ANOVA P	0.060	0.160	0.001	0.074	0.093	0.005	0.018	0.035	NA	NA

^s^Shoot height data for trial 3 not presented because no strains caused an increase compared to the control based on Dunnett's test and there was no significant treatment effect in the ANOVA. ^t^Percentage increase of a growth variable by bacterial treatment compared to the control. ^u^GSF = growth stimulation frequency; frequency at which a strain increased growth (at 95% confidence level) in all measurements across trials. ^v^FIT2 = frequency in top 2 strains category. ^w^Numbers followed by the same letter in each column are not significantly different at *α* = 0.05 according to LSD test. ^x^NA = not applicable. ^*∗*^Significant difference between treatment and control (*P* ≤ 0.05) according to Dunnett's test. — = no data because strain was not tested

**Table 6:** Profile of *in vitro* physiological traits exhibited by *Bacillus* strains with high and low effectiveness in promoting corn growth as determined in greenhouse pot experiments.

Strain	Antib	Antif	Pro	Bios	Sid	Phos	IAA	Pouch assay	Efficacy on corn
*B. megaterium* R181	+	+	+	−	+	+	+	+	High
*B. safensis* R173	−	−	+	+	−	+	+	−	
*B. safensis* R176	−	−	+	+	−	−	+	+	
*B. simplex* R180	−	−	+	−	+	−	−	+	
*Paenibacillus cineris* R177	−	−	−	−	−	+	+	+	
*P. graminis* R200	−	−	−	−	−	−	−	+	

*Bacillus acidiceler* R228	−	−	+	−	−	−	+	+	Low
*B. megaterium* R232	−	−	+	−	+	+	+	+	
*B. pumilus* R174	−	+	+	+	−	−	−	+	
*B. pumilus* R183	+	+	+	+	−	−	−	−	
*B. pumilus* R190	+	+	+	+	+	−	−	−	
*Lysinibacillus fusiformis* R198	−	−	−	−	−	−	+	+	

Antib = antibacterial inhibition; Antif = antifungal inhibition; Pro = protease activity; Bios = biosurfactant production; Sid = siderophore production; Phos = phosphate solubilization; IAA = indole acetic acid; pouch assay = promotion of corn growth in growth pouches. + (shaded) = trait exhibited; − = trait absent. *Note.* Chitinase and nitrogen fixation results are not shown because these traits were negative for all strains.

**Table 7:** Effects of *Bacillus* test strains on corn growth in three growth pouch experiments (shoot height and shoot weight data not available for trial 3).

Strain	% increase compared to control^x^									
	Lateral root number			Root length (cm)			Shoot height (cm)		Shoot fresh weight (g)	
	T1^y^	T2	T3	T1	T2	T3	T1	T2	T1	T2
*Bacillus acidiceler* R228	17	20	0	27	15^*∗*^	10	44^*∗*^	18^*∗*^	40^*∗*^	20
*B. megaterium* R181	50^*∗*^	20	−10	41^*∗*^	−17	8	0	13	0	20
*B. megaterium* R232	17	40^*∗*^	−10	23	2	4	39^*∗*^	9	40^*∗*^	20
*B. pumilus* R174	33^*∗*^	20^*∗*^	−10	37^*∗*^	2	10	28^*∗*^	23^*∗*^	20	20
*B. pumilus* R183	17	−40	−20	46^*∗*^	−17	−4	−6	−9	−20	0
*B. pumilus* R190	−17	−20	−10	10	−4	−2	17	−5	20	0
*B. safensis* R173	−17	0	0	23	−18	8	−11	27^*∗*^	−20	20
*B. safensis* R176	−50	40^*∗*^	−10	−10	−17	10	22^*∗*^	18	20	20
*B. simplex* R180	17	0	−10	46^*∗*^	−7	14	22^*∗*^	13	20	20
*Lysinibacillus fusiformis* R198	33^*∗*^	−20	−10	18	−10	20^*∗*^	28^*∗*^	32^*∗*^	20	40^*∗*^
*Paenibacillus cineris* R177	33^*∗*^	−20	−10	37^*∗*^	−13	2	11	13	0	20
*P. graminis* R200	33^*∗*^	0	0	41^*∗*^	−15	14	22^*∗*^	18	40^*∗*^	20

^x^Percentage increase of a growth variable by bacterial treatment compared to the control. ^y^T = trial. ^*∗*^Significant difference between treatment and control (*P* ≤ 0.05) according to Dunnett's test.

## Data Availability

The data used to support the findings of this study are included as tables within the article. Additionally, sequence data were submitted to GenBank, and accession numbers are included within this article. Anyone needing additional information on any aspect of the data should contact the corresponding author (tony.adesemoye@unl.edu).
